# Corticosteroids in treatment of aspiration-related acute respiratory distress syndrome: results of a retrospective cohort study

**DOI:** 10.1186/s12890-016-0194-4

**Published:** 2016-02-10

**Authors:** Jiang-nan Zhao, Yao Liu, Huai-chen Li

**Affiliations:** Department of Respiratory Medicine, The First Hospital of Jiaxing, Jiaxing, China; Department of Respiratory Medicine, Shandong Provincial Hospital Affiliated to Shandong University, Jinan, Shandong 250021 China

## Abstract

**Background:**

Acute stroke patients suffering from aspiration may present with acute respiratory distress syndrome (ARDS). There is still a lack of convincing data about the efficacy of corticosteroids in the treatment of aspiration-related ARDS. Therefore, we evaluated the clinical impact of corticosteroids on aspiration-related ARDS.

**Methods:**

Between 2012 and 2014, we conducted a retrospective study among acute stroke patients diagnosed with aspiration-related ARDS. The data analyzed included demographic characteristics, clinical manifestations, laboratory examinations, chest imaging, and hospital discharge status.

**Results:**

Seventy-three acute stroke patients were diagnosed with aspiration-related ARDS. The hospital mortality rate was 39.7 %. Corticosteroids were administered in 47 patients (64.4 %). The mean dosage was 1.14 (standard deviation [SD] 0.47) mg/kg daily of methylprednisolone (or an equivalent) by intravenous infusion for a period of 7.3 (SD 3.8) days. Ground glass opacities in chest computed tomography images were resolved when corticosteroids were administered. The admission National Institute of Health Stroke Scale score (odds ratio [OR] 5.17, 95 % confidence interval [CI] 1.27–10.64) and Acute Physiology and Chronic Health Evaluation II score (OR 2.00, 95 % CI 1.12–3.56) were associated with an increased risk of hospital mortality, while albumin (OR 0.81, 95 % CI 0.64–0.92) and corticosteroids therapy (OR 0.50, 95 % CI 0.35–0.70) were associated with a decreased risk.

**Conclusions:**

Low-dose and short-term corticosteroid therapy may have an impact on survival in aspiration-related ARDS. The presence of ground glass opacities on the chest computed tomography, performed to rule out aspiration-related ARDS, could be translated into an increased possibility of positive response to corticosteroid therapy.

## Background

Aspiration of oropharyngeal or gastric contents flowing into the lower respiratory tract may result in several pulmonary diseases [1–3], such as airway obstruction, aspiration lung abscess, aspiration pneumonia, aspiration pneumonitis, and even acute respiratory distress syndrome (ARDS) [3, 4]. Among stroke patients, ARDS caused by aspiration is a major cause of death. The early diagnosis of aspiration-related ARDS is crucial to improve patient outcomes, as well as to choose the optimal treatment, including mechanical ventilation settings [5, 6]. Little is known about the clinical features and outcomes of aspiration-related ARDS.

Because dysregulated inflammation is the cardinal feature of ARDS [7, 8], it seems to be a rational choice to use corticosteroids as part of the treatment. In the early 1980s, clinical investigators found that the inflammatory exudation in patients with ARDS could be decreased with systemic corticosteroid therapy [9]. Meduri and colleagues found that peripheral blood leukocytes, which are exposed to plasma from patients with ARDS, can produce inflammatory cytokines. If methylprednisolone was given to these patients, the inflammation reaction could be reduced [10]. Conversely, Sukumaran and colleagues found that patients who were given corticosteroids experienced longer stay in the intensive care unit. However, there were no significant differences in the outcome [11]. Moreover, in a case–control study, patients treated with corticosteroids were more easily infected by gram-negative bacteria and developed pneumonia more frequently [12].

In current clinical practice, systemic corticosteroids are often used as part of the treatment of aspiration-related ARDS, depending on the discretion of individual pulmonologist. However, the effect of systemic corticosteroids on the prognosis of the disease has not yet been revealed. Therefore, we conducted a retrospective study of acute stroke patients with aspiration-related ARDS aiming to evaluate the efficacy of corticosteroids and to identify predictors of hospital mortality.

## Methods

The study was approved by the Ethics Committee of Shandong Provincial Hospital, Shandong Province, China. Patient records were anonymized and de-identified prior to analysis.

Our study was a retrospective single-center study that included about 2986 consecutive acute stroke patients admitted to the adult medical neurology department between 1 January 2012 and 31 July 2014. All patients were selected at the Shandong Provincial Hospital, a tertiary-care, university-affiliated hospital. To be eligible, all acute stroke patients had to meet all of the following criteria [13, 14]: (1) aged 18 years or older; (2) diagnosed with rapidly developed clinical signs of cerebral function disturbance of vascular origin, and classified based on results from the first brain scan into cerebral infarct, intracerebral hemorrhage, and subarachnoid hemorrhage, according to the World Health Organization definition; (3) presented within 24 h of the onset of acute stroke; (4) confirmed by head computerized tomography (CT) or brain magnetic resonance imaging (MRI).

### Inclusion criteria

For inclusion, the following clinical signs and symptoms after the episode of aspiration had to be present: (1) a subjective worsening of dyspnea, development of hypoxia with pulse oxygen saturation (SPO2) < 90 mmHg, radiographic pulmonary abnormalities presented by chest radiography or CT that were reviewed by two radiologists; (2) or abnormal breath sounds, fever (≥37.5 °C) or leukocytosis; (3) or requirement for intensive care (defined as the use of mechanical ventilation or the need for treatment with vasopressors against shock). To be included, all patients had to conform to the first item. Either the aspiration (following an episode of dysphagia, choking, vomiting or regurgitation) had been observed, or gastric contents had been suctioned from the endotracheal tube following intubation. Although, it is difficult to monitor the occurrence of aspiration, if one person with healthy lungs showed the above mentioned clinical symptoms, we considered aspiration as the root cause. After the diagnosis of aspiration-related lung injury, all patients were managed by both neurologists and pulmonologists.

The diagnosis of ARDS was established by the treating physician, and it was based on the Berlin definition [8]. Aspiration-related ARDS was defined as ARDS developed after aspiration. Other etiologies of ARDS such as sepsis, major trauma, multiple transfusions, pulmonary contusion, and acute pancreatitis were excluded. Conditions in which patients frequently have hypoxia and diffuse pulmonary infiltration, such as pulmonary edema, congestive heart failure, interstitial lung disease, active tuberculosis, radiation pneumonitis, pulmonary infiltration with eosinophilia, widespread infection, pulmonary alveolar hemorrhage alveolar proteinosis, bronchioloalveolar cell carcinoma were also ruled out.

### Exclusion criteria

Patients with the following conditions were excluded: nosocomial pneumonia; severe immunosuppression (Acquired Immune Deficiency Syndrome (AIDS), use of immuno-suppressant such as cytotoxic drugs, cyclosporine, monoclonal antibodies, among others); preexisting medical condition with life expectancy lower than 3 months (i.e., malignancy); pregnancy; major gastrointestinal bleeding (GIB) within 3 months of the current hospitalization; acute asthma, chronic obstructive pulmonary disease or autoimmune disorders (i.e., any condition requiring more than 0.5 mg/kg/day of prednisone equivalent); and hepatic cirrhosis.

### Data collection

For the present study, the following data were analyzed: age, sex, National Institutes of Health Stroke Scale (NIHSS) score on admission, Glasgow Coma Scale (GCS) score on admission, smoking history, excess alcohol consumption (≥2 standard alcohol beverages per day), preexisting comorbidities (hypertension, diabetes, coronary heart disease, liver disease, kidney disease, among others), clinical symptoms on admission and new symptoms and signs after admission, PaO_2_/FiO_2_, mechanical devices (invasive/non-invasive mechanical ventilation used after admission for unsolved hypoxia despite of high-flow oxygen), time from diagnosis to corticosteroid therapy, chest radiological findings, and Acute Physiology and Chronic Health Evaluation II (APACHE II) score for patients with ARDS, laboratory indices (routine blood counts, liver function indicators, routing chemistry tests laboratory, blood gas analysis, and others), hospital length-of-stay (LOS) (days), hospital discharge status (survivor, dead). All data were extracted from the electronic medical record (EMR) system by trained research coordinators.

### Corticosteroid therapy

The dose and administration intervals of corticosteroid therapy were collected from EMR records. Patient response to treatment and outcome were evaluated by the following criteria: decrease in respiratory rate (≤20/min), increase in oxygenation at rest and sleep (SPO_2_ ≥ 90 mmHg), and improvement in chest CT images.

The commonly expected adverse events included new infection, hyperglycemia with additional glucose-lowering therapy, GIB, and neuromyopathic complications. A new infection was defined as an infection, not present or incubating before the administration of corticosteroids. Infections sites included pulmonary, blood, urinary, skin wound and other organs/tissues. Pulmonary infection was indicated by newly emerging increase in temperature, leukocytosis, radiological abnormalities, and decline in PaO_2_ compared with the initial observations (before the administration of corticosteroid therapy).

### Statistical analysis

Continuous variables were summarized with mean and standard deviation (SD); categorical variables were summarized as proportions. In univariate analysis, a *X*^*2*^ test was used to compare categorical variables, and a *t* test with equal variance was used to compare continuous variables. To identify independent factors that were associated with hospital mortality, multivariate logistic regression analysis was used. The adjusted odds ratios (OR) and the 95 % confidence interval (CI) and *P* values for individual variables were obtained using a logistic regression model. *P* < 0.05 was considered statistically significant. All analyses were performed with SPSS version 17.0 for Windows (SPSS Inc., Chicago, IL, USA).

## Results

### Patient population

Of the 2286 acute stroke patients enrolled in the study, 551 patients were diagnosed with aspiration pneumonia. According to the inclusion and exclusion criteria, 73 patients were diagnosed with aspiration-related ARDS. Patients’ clinical characteristics are shown in Table 1. Of these, 52 patients were men, and the mean age was 67 (SD 14.1) years. The mean NIHSS and GCS scores were 9.3 (SD 3.2) and 5.6 (SD 2.0), respectively. The mean hospital LOS was 23 (SD 7.0) days.

**Table 1:** Baseline characteristics of enrolled patients

Characteristics	Total (n, %)
Number	73
Age (years)	67.0 (SD 14.1)
Sex male	52 (71.2)
Admission NIHSS score	9.3 (SD 3.2)
Admission GCS score	5.6 (SD 2.0)
Smoking	30 (41.1)
Excess alcohol consumption	22 (30.1)
Pre-existing illnesses	
Hypertension	53 (72.6)
Diabetes	36 (49.3)
Coronary heart disease	14 (19.2)
Liver disease (except for hepatic cirrhosis)	0 (0)
Kidney disease	2 (2.7)
Chest X ray	73 (100)
Chest CT	58 (79.5)
GGOs	40/58 (69.0)
Consolidation opacities	31/58 (53.4)
Pleural effusions	14/58 (24.1)
Air bronchogram	11/58 (19.0)
Tachypnea (RR ≥ 25 breaths/min)	67 (91.8)
Fever (T ≥ 37.5 °C)	43 (58.9)
PaO_2_/FiO_2_ (mmHg)	143.5 (SD 59.0)
APACHE II score	28.2 (SD 10.4)
pH value	7.38 (SD 0.57)
Acidosis (pH < 7.35)	11 (15.1)
Albumin (g/L)	26.1 (SD 9.2)
Treatment	
Corticosteroids	47 (64.4)
No respiratory response	16/47 (34.0)
Respiratory response	31/47 (66.0)
Ventilatory support	63 (86.3)
Non-invasive ventilation alone	14/63 (22.2)
Non-invasive ventilation followed by intubation	16/63 (25.4)
First line invasive ventilation	33/63 (52.4)
Time from diagnosis to corticosteroids (days)	6.5 (SD 3.5)
Outcomes	
Duration of IMV (days)	8.5 (SD 5.5)
Hospital LOS (days)	23.0 (SD 7.0)
Hospital mortality	29 (39.7)

*Abbreviation*: *SD* standard deviation, *NIHSS* National Institutes of Health Stroke Scale, *GCS* Glasgow Coma Scale, *GGOs* ground glass opacities, *RR* respiratory rate, *APACHE* Acute Physiology and Chronic Health Evaluation, *PaO*_*2*_ partial pressure of oxygen in arterial blood, *FiO*_*2*_ fraction of inspired oxygen, *IMV* invasive mechanical ventilation, *LOS* length-of-stay

Of the 73 patients with aspiration-related ARDS, mechanical ventilation (MV) was required in 63 patients, including invasive MV (IMV) (*n* = 49, 77.8 %) and non-invasive MV (*n* = 14, 22.2 %). According to the EMR records, the surrogates of 10 patients with aspiration-related ARDS refused to use MV care and signed the informed consent for refusing treatment. The mean duration of IMV was 8.5 (SD, 5.5) days. In total, 29 patients died with a mortality rate of 39.7 %.

### Corticosteroid therapy

**Fig. 1:**
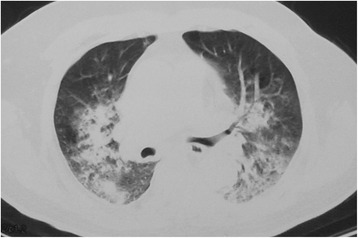
Radiologic finding in a 78-year-old cerebral infarct man with aspiration-related ARDS. Chest CT showed ground-glass opacities, inhomogenous patchy consolidations and pleural effusion in bilateral lobes

**Fig. 2:**
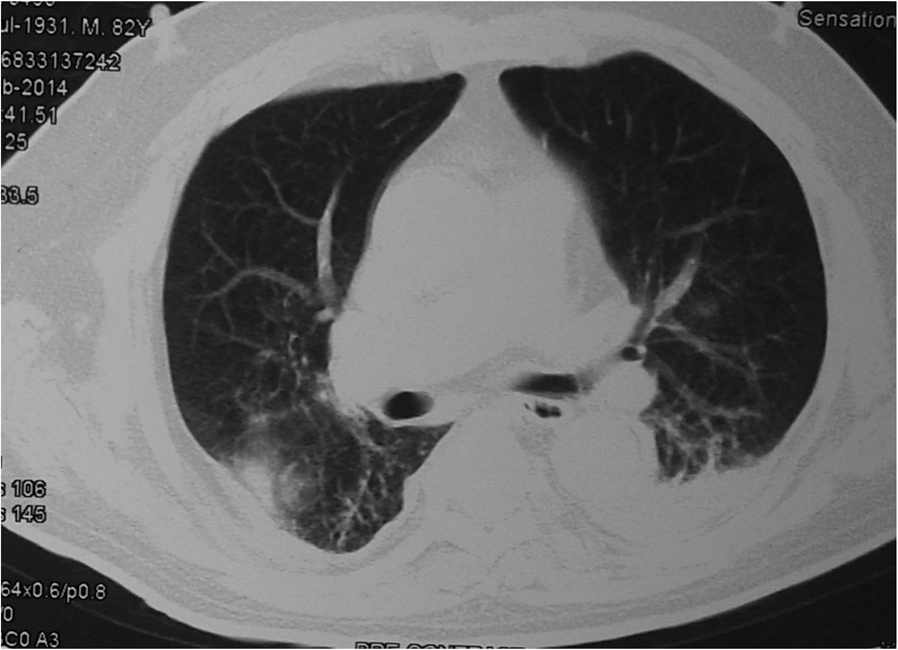
The patient (weight = 73 kg) was treated with intravenous methylprednisolone (80 mg/day for 8 consecutive days). Chest CT scan demonstrated decreased density and extent of pulmonary opacification involving both lungs

Corticosteroid therapy was used in 47 (64.4 %) patients after the onset of aspiration-related ARDS. On average, it took 6.5 (SD 3.5) days from ARDS diagnosis to corticosteroid therapy initiation. Of the 47 patients, 31 (66 %) met the criteria for responsiveness (Figs. 1 and 2). Among the 21 patients who underwent IMV who responded to corticosteroid therapy, 17 patients were successfully extubated within 15 days. The remaining four patients experienced fatal complications. The corticosteroid dose ranged from 20 mg/day to 160 mg/day of methylprednisolone (or its equivalent), and the duration ranged from 2 to 17 days. The mean daily dose of methylprednisolone (or its equivalent) was 1.14 (SD 0.47) mg/kg, and the mean duration was 7.3 (SD 3.8) days.

By univariate analysis, the proportion of patients with GGOs in chest images differed significantly (*P* < 0.05) in the responsive and nonresponsive groups (Table 2). There was no significant difference in any adverse event between two groups. The adverse events of patients treated with and without systemic corticosteroids are shown in Table 3.

**Table 2:** Univariate analyses of factors associated with corticosteroids responsiveness

	Response (*n* = 31,%)	Non-response (*n* = 16,%)	*P* value
Age (years)	65.8 (SD 9.5)	67.1 (SD 14.2)	0.21
Sex male	21 (67.4)	13 (68.4)	0.49
NIHSS score	9.0 (SD 3.2)	9.3 (SD 2.8)	0.50
GCS score	6.0 (SD 2.1)	5.8 (SD 2.1)	0.72
Smoking	12 (38.7)	7 (43.6)	0.76
Excess alcohol consumption	9 (29.0)	5 (31.3)	1.00
Pre-existing illnesses			
Hypertension	22 (71.0)	11 (68.9)	0.90
Diabetes	16 (51.6)	8 (50.0)	0.17
Coronary heart disease	6 (19.4)	3 (18.9)	1.00
Chest CT			
GGOs	23 (74.2)	7 (43.8)	0.04
Consolidation opacities	16 (51.6)	9 (56.3)	1.00
Pleural effusions	10 (32.3)	4 (25.0)	0.74
Air bronchogram	6 (19.4)	4 (25.0)	0.72
PaO_2_/FiO_2_ (mmHg)	139.5 (SD 55.5)	147.0 (SD 63.0)	0.99
APACHE II score	27.4 (SD 13.6)	28.4 (SD 11.1)	0.54
pH value	7.40 (SD 0.6)	7.38 (SD 0.5)	0.41
Albumin (g/L)	26.6 (SD 7.9)	27.9 (SD 8.3)	0.88
Time from diagnosis to corticosteroids (days)	5.5 (SD 2.0)	6.0 (SD 2.5)	0.16

*Abbreviation*: *SD* standard deviation, *NIHSS* National Institutes of Health Stroke Scale, GCS Glasgow Coma Scale, *GGOs* ground glass opacities, *APACHE* Acute Physiology and Chronic Health Evaluation, *PaO*_*2*_ partial pressure of oxygen in arterial blood, *FiO*_*2*_ fraction of inspired oxygen

**Table 3:** Adverse events between patients with and without systemic corticosteroids

	Systemic corticosteroids (*n* = 47, %)	Without corticosteroids (*n* = 26, %)	*P* value
Hyperglycemia with additional glucose-lowering therapy	17 (36.2)	7 (26.9)	0.45
New infection	13 (27.7)	6 (23.1)	0.78
GIB	1 (2.1)	0 (0)	1.00

*Abbreviation*: *GIB* gastrointestinal bleeding

### Risk factors of hospital mortality

The 10 patients, whose surrogates refused MV, were excluded from the analysis. By univariate analysis, age, male sex, admission NIHSS score, excess alcohol consumption, hypertension, GGOs in chest CT images, PaO_2_/FiO_2_, APACHE II score, albumin, corticosteroid therapy with response, and IMV support were significantly (*p* values < 0.05) associated with hospital mortality.

The significant risk factors for hospital mortality according to multiple logistic regression analysis are shown in Table 4. The analysis showed that the protective roles of hospital mortality were albumin (OR 0.81, 95 % CI 0.64–0.92) and corticosteroid therapy (OR 0.50, 95 % CI 0.35–0.70). The two factors that were independently associated with hospital mortality were admission NIHSS score (OR 5.17, 95 % CI 1.27–10.64) and APACHE II score (OR 2.00, 95 % CI 1.12–3.56).

**Table 4:** Determinants of hospital mortality

	Univariate analysis		Multivariate analysis	
	OR (95 % CI)	*P* value	OR (95 % CI)	*P* value
Age	0.99 (0.98–1.00)	0.11		
Sex male	1.36 (1.17–1.59)	<0.001		
NIHSS score	4.01 (2.98–5.56)	<0.001	5.04 (1.56–16.28)	0.007
GCS score	0.94 (0.82–1.07)	0.36		
Smoking	1.30 (0.86–1.99)	0.22		
Excess alcohol consumption	1.55 (1.27–1.88)	<0.001		
Pre-existing illnesses				
Hypertension	1.18 (1.01–1.39)	0.03		
Diabetes	1.00 (0.95–1.06)	0.89		
Coronary heart disease	1.09 (0.85–1.67)	0.31		
Kidney disease	1.29 (0.52–3.19)	0.59		
Chest CT				
GGOs	0.49 (0.27–0.86)	0.01		
Consolidation opacities	2.00 (0.92–3.56)	0.09		
Pleural effusions	1.05 (0.65–5.22)	0.25		
Air bronchogram	1.91 (0.54–6.84)	0.32		
PaO_2_/FiO_2_	0.19 (0.05–0.82)	0.03		
APACHE II score	2.22 (1.90–2.58)	<0.001	1.51 (1.05–2.16)	0.03
pH value	0.99 (0.30–3.31)	0.99		
Albumin	0.94 (0.88–1.00)	0.08	0.88 (0.79–0.97)	0.001
Treatment				
Corticosteroids	0.33 (0.23–0.47)	<0.001	0.50 (0.35–0.70)	<0.001
Non-invasive ventilation	1.69 (0.74–3.88)	0.21		
Invasive ventilation	0.06 (0.01–0.59)	0.02		
Time from diagnosis to corticosteroids treatment	1.28 (0.90–1.82)	0.16		

*Abbreviation*: *SD* standard deviation, *NIHSS* National Institutes of Health Stroke Scale, *GCS* Glasgow Coma Scale, *GGOs* ground glass opacities, *APACHE* Acute Physiology and Chronic Health Evaluation, *PaO*_*2*_ partial pressure of oxygen in arterial blood, *FiO*_*2*_ fraction of inspired oxygen

## Discussion

Acute respiratory distress syndrome causes severe acute respiratory failure with dynamic impairment in oxygen and carbon dioxide transfer, and it is associated with the need for mechanical ventilation to provide high levels of supplementary oxygen [7, 8]. Given the impaired consciousness and swallowing difficulty, acute stroke patients are at great risk for aspiration [14, 15]. Patients who have aspirated gastric material may present with dramatic signs and symptoms, such as gastric material in the oropharynx as well as wheezing, coughing, dyspnea, cyanosis, pulmonary edema, and hypoxia. Any of these signs or symptoms could rapidly progress to ARDS [3]. Until now, no study has comprehensively described the clinical characteristics and outcomes of aspiration-related ARDS. After the first few victims were encountered at our institution, we became alert to patients with similar clinical courses because of their bad prognosis and high mortality rate.

In the present study, we found a high hospital mortality rate of 39.7 % for stroke patients with aspiration-related ARDS, among whom 86.3 % underwent MV, including IMV and non-invasive MV. As dysregulated inflammation is the cardinal feature of ARDS, corticosteroids are often added to the treatment based on the discretion of the treating pulmonologist. In our retrospective study, we found that corticosteroids therapy was administered in about two-thirds of patients, among which 66 % responded to the treatment. Patients with GGO pattern in chest CT images responded better to corticosteroid therapy than those who did not present that radiologic pattern. Admission NIHSS score, APACHE II score, albumin, and corticosteroid therapy were associated with hospital mortality.

Diagnosing aspiration-related ARDS is a major challenge. Rales on auscultation, tachypnea, and fever are nonspecific signs that cannot be used to identify patients. Conversely, chest CT images are more sensitive for diagnosing ARDS than chest radiographs [16–18]. We found that diffuse GGOs evidenced in CT images had a favorable response to corticosteroid therapy. The GGOs were defined as increased pulmonary attenuation, with preserved bronchial and vascular margins. We consider it advisable to perform chest CT timely in order identify aspiration-related ARDS early and to promptly apply proper treatment with the aim of improving the prognosis of our patients. The presence of a GGO pattern can alert the physician that this patient may respond positively to corticosteroid therapy. Thus, a careful assessment of physical symptoms and signs and chest CT images is crucial.

Excessive and protracted inflammation is the pathophysiological basis of ARDS, which would result in multi-organ dysfunction and even death [7, 19, 20]. Corticosteroids are added to the medications because of their important role as anti-inflammatory elements. However, the role of corticosteroids in managing ARDS remains uncertain because of insufficient scientific evidence to provide clinicians with clear and robust guidance [9, 10, 12, 21]. Although existing studies were unable to provide clear evidence of the benefits or harmful effects of corticosteroids, some studies pointed out that patients would benefit patients from steroid administration after the onset of ARDS, particularly to reduce mortality [11]. A systematic review and meta-analysis showed that the use of low-dose corticosteroids was associated with improved mortality and morbidity outcomes without notable side effects [22].

In the present study, corticosteroid therapy was administered in 64.6 % of patients with aspiration-related ARDS, among which 66.0 % responded to the treatment. The mean daily dose of methylprednisolone (or its equivalent) was 1.14 (SD 0.47) mg/kg, and the mean duration of treatment was 7.3 (SD 3.8) days. Based on our results, corticosteroid therapy was an independent protective factor for hospital mortality, which suggests that low-dose and short-term corticosteroid use might indeed be beneficial for patients with aspiration-related ARDS. Response to corticosteroid therapy, whether it is ineffective, effective, or toxic, is influenced by the dosage used and duration of the administration [23–25]. The clinical side effects of systemic corticosteroids should not be ignored, such as new infection, hyperglycemia and GIB. One major concern is the high risk of infection, especially aspiration induced lung abscess, secondary to immunosuppression. The adverse events often lead to undesired results. High-dose and long-term corticosteroid therapy may cause serious side effects. In our study, the occurrence of adverse events did not differ between the patients treated with and without the low-dose and short-term corticosteroid schedule. Consequently, in future studies to elucidate the role of corticosteroids in aspiration-related ARDS, it should be taken into account not only which subset of patients can potentially benefit from its administration, but also the optimal dose and duration of corticosteroid therapy. Only in this way, we can achieve the balance between the beneficial and harmful effects of the inflammatory response.

The high mortality observed in this study was also associated with the severity of the illness, which was reflected by NIHSS scores and APACHE II score. Another predictor of hospital mortality was serum albumin, which regulates plasma osmotic pressure. Hypoproteinemia accelerates fluid exudation, promotes alveolar edema, and contributes to ventilation-perfusion imbalance [26]. These suggest that early diagnosis combined with early treatment will benefit the outcome.

Some limitations of this study should be mentioned. First, the analysis was retrospective. The clinical practice and predictive factors of mortality could change substantially. Moreover, we used a single-center design, and the number of patients studied was limited. It is necessary to conduct further multicenter prospective studies to reach more accurate conclusions. Second, the dosage and term of systemic corticosteroids varied for each patient. Timing of corticosteroid administration might also play a critical role in the effects of treatment because the inflammatory response is a dynamic process. Third, adrenocortical function was not evaluated in our study. Elderly stroke patients tend to have a relative adrenal insufficiency [27, 28]. Therefore, the possibility of systemic corticosteroid compensation for adrenal insufficiency should be considered.

## Conclusions

Low-dose, short-term corticosteroid therapy may be expected to be effective in reduce hospital mortality in cases of aspiration-related ARDS, without notable side effects. The presence of a GGO pattern in chest CT images obtained in cases of suspected aspiration-related ARDS could translate into an increased possibility of positive response to corticosteroid therapy. However, we are unable to reach an accurate conclusion in terms of defining the optimal dose, timing, and duration of corticosteroid therapy. Definitive treatment recommendations will depend on further larger-scale, randomized, controlled prospective trials.

## Abbreviation

AIDS: acquired Immune Deficiency Syndrome
APACHE: Acute Physiology and Chronic Health Evaluation
ARDS: acute respiratory distress syndrome
CI: confidence interval
COPD: chronic obstructive pulmonary disease
CT: computerized tomography
EMR: electronic medical record
FiO_2_: fraction of inspired oxygen
GCS: Glasgow Coma Scale
GGOs: ground glass opacities
GIB: gastrointestinal bleeding
IMV: invasive mechanical ventilation
LOS: length-of-stay
MRI: magnetic resonance imaging
NIHSS: National Institutes of Health Stroke Scale
OR: odds ratios
PaO_2_: partial pressure of oxygen in arterial blood
RR: respiratory rate
SD: standard deviation
SPO_2_: pulse oxygen saturation
